# Supplementary material for: Vaccination discourses among chiropractors, naturopaths and homeopaths: A qualitative content analysis of academic literature and Canadian organizational webpages
Source: PLoS One. 2020 Aug 12;15(8):e0236691. doi: 10.1371/journal.pone.0236691 (PMC7423113; doi:10.1371/journal.pone.0236691)
Supplement: S1 Table — (DOCX) [file pone.0236691.s001.docx]

| Database | Date searched | Search terms | Filters | Results (# items) |
| --- | --- | --- | --- | --- |
| PubMed | October 9, 2018 | ((booster OR boosters OR chemoprophylaxis OR immunization OR immunizations OR immunizing OR immunology OR immunoprevention OR immunotherapeutics OR immunotherapies OR immunotherapy OR nanovaccination OR nanovaccine OR nanovaccines OR prophylaxis OR vaccination OR vaccinations OR vaccine OR vaccines OR vaccinology OR “vaccine-induced immunity”) AND ((chiropractic OR chiropractor OR chiropractors OR “doctors of chiropractic” OR manipulation, chiropractic) OR (ethnobotany OR holism OR “natural medicine” OR “nature cure” OR naturism OR naturopath OR naturopathic OR naturopaths OR naturopathy) OR (“anthroposophic medicine” OR “anthroposophical medicine” OR homeopath OR homeopathic OR homeopaths OR homeopathy OR isopathic OR isopathy)) | Article type: all  Text availability: all  Publication dates: all  Sort by: most recent | 1339 |
| Embase | November 9, 2018 | (exp vaccination/ or vaccination.mp. or exp vaccination coverage/ or exp vaccination reaction/ or exp vaccination refusal/ or exp vaccine/ or vaccine.mp. or exp vaccine failure/ or exp vaccine immunogenicity/ or exp immunization/ or immunization.mp. or exp immunology/ or immunology.mp.) and (exp chiropractic/ or chiropractic.mp. or exp chiropractic education/ or exp chiropractic manipulation/ or exp chiropractic practice/ or exp chiropractic student/ or exp chiropractor/ or chiropractor.mp. or exp manipulative medicine/ or manipulative medicine.mp. or (exp alternative medicine/ or naturopath.mp. or naturopathy.mp. or naturopathic.mp. or natural medicine.mp.) or (exp homeopathic agent/ or exp homeopathy/ or homeopathy.mp. or homeopath.mp. or homeopathic.mp. or homeopaths.mp. or anthroposophic.mp. or anthroposophical.mp. or isopathic.mp. or isopathy.mp.)) | Years: all  Subject: all  Author: all  Journal: all  Publication type: all | 1471 |
| PsycINFO | November 11, 2018 | **Any Field**: vaccination *OR* **Any Field**: vaccinations *OR* **Any Field**: vaccine *OR* **Any Field**: vaccines *OR* **Any Field**: immunization *OR* **Any Field**: immunizations *OR* **Any Field**: immunizing *OR* **Any Field**: immunology *OR* **Any Field**: immunotherapy *OR***Any Field**: immunotherapeutics *OR* **Any Field**: nanovaccine *OR* **Any Field**: nanovaccines *OR* **Any Field**: nanovaccination *OR* **Any Field**: prophylaxis *OR* **Any Field**: immunoprevention *OR* **Any Field**: booster *OR* **Any Field**: chemoprophylaxis *OR* **Any Field**: vaccinology *AND* **Any Field**: naturopathy *OR* **Any Field**: naturopathic *OR* **Any Field**: ethnobotany *OR* **Any Field**: holism *OR* **Any Field**: naturism *OR* **Any Field**: “natural medicine” *OR* **Any Field**: “nature cure” *OR* **Any Field**: naturopath *OR* **Any Field**: naturopaths*OR* **Any Field**: anthroposophical *OR* **Any Field**: anthroposophy *OR* **Any Field**: homeopath *OR* **Any Field**: homeopaths *OR* **Any Field**: homeopathic *OR* **Any Field**: homeopathy *OR* **Any Field**: isopathic *OR* **Any Field**: isopathy *OR* **Any Field**: chiropractic *OR* **Any Field**: “chiropractic manipulation” *OR* **Any Field**: chiropractor *OR* **Any Field**: chiropractors *OR* **Any Field**: “doctor of chiropractic” *OR* **Any Field**: “complementary medicine” *OR* **Any Field**: “alternative medicine” *OR* **Any Field**: “complementary and alternative medicine” | Filters: none | 140 |
| Cochrane | November 11, 2018 | (vaccine OR vaccines OR vaccination OR vaccinations OR immunization OR immunizations OR immunotherapy OR immunizing OR immunology OR immunotherapies OR immunotherapeutics OR nanovaccines OR nanovaccine OR nanovaccination OR prophylaxis OR chemoprophylaxis OR immunoprevention OR booster OR vaccinology) AND (chiropractic OR chiropractor OR chiropractors OR “chiropractic manipulation” OR “doctor of chiropractic” OR homeopathy OR homeopathic OR homeopath OR homeopaths OR isopathic OR isopathy OR anthroposophical OR anthroposophic OR naturopathy OR naturopath OR naturopaths OR naturopathic OR ethnobotany OR holism OR “natural medicine” OR “nature cure”) | Filters: none  Order by: relevancy | 57 |
| CINAHL | November 12, 2018 | (vaccination OR vaccinations OR vaccine OR vaccines OR immunization OR immunizations OR booster OR immunotherapy OR immunotherapies OR immunotherapeutics OR immunoprevention OR nanovaccine OR nanovaccines OR nanovaccination OR prophylaxis OR chemoprophylaxis OR “vaccine-induced immunity” OR vaccinology ) AND ( naturopathy OR naturopathic OR ethnobotany OR holism OR naturism OR “natural medicine” OR “nature cure” OR naturopath OR naturopaths OR anthroposophical OR anthroposophy OR homeopath OR homeopaths OR homeopathic OR homeopathy OR isopathic OR isopathy OR chiropractic OR “chiropractic manipulation” OR chiropractor OR chiropractors OR “doctor of chiropractic” OR “complementary medicine” OR “alternative medicine” OR “complementary and alternative medicine” ) | Filters: none  Order by: relevancy | 411 |
| Web of Science | November 12, 2018 | [TS=(vaccination OR vaccinations OR vaccine OR vaccines OR immunization OR immunizations OR booster OR immunotherapy OR immunotherapies OR immunotherapeutics OR immunoprevention OR nanovaccine OR nanovaccines OR nanovaccination OR prophylaxis OR chemoprophylaxis OR “vaccine-induced immunity” OR vaccinology)] AND [TS= (naturopathy OR naturopathic OR ethnobotany OR holism OR naturism OR “natural medicine” OR “nature cure” OR naturopath OR naturopaths OR anthroposophical OR anthroposophy OR homeopath OR homeopaths OR homeopathic OR homeopathy OR isopathic OR isopathy OR chiropractic OR “chiropractic manipulation” OR chiropractor OR chiropractors OR “doctor of chiropractic” OR “complementary medicine” OR “alternative medicine” OR “complementary and alternative medicine”)] | Filters: none  Sort by: date | 472 |
