# Supplementary material for: Vaccination discourses among chiropractors, naturopaths and homeopaths: A qualitative content analysis of academic literature and Canadian organizational webpages
Source: PLoS One. 2020 Aug 12;15(8):e0236691. doi: 10.1371/journal.pone.0236691 (PMC7423113; doi:10.1371/journal.pone.0236691)
Supplement: S2 Table — (DOCX) [file pone.0236691.s002.docx]

| URL | Organization name | Year of publication or date last revised |
| --- | --- | --- |
| Chiropractic organizations | | |
| <https://www.chiropractic.ca/about-cca/code-of-ethics/vaccination-immunization/> | Canadian Chiropractic Association | 2019 |
| <https://www.chiropractic.on.ca/members/about-oca/position-statements/immunization-and-vaccination/> | Ontario Chiropractic Association | 2017 |
| <http://www.bcchiro.com/> | British Columbia Chiropractic Association | 2018 |
| <https://albertachiro.com/> | Alberta College & Association of Chiropractors | 2016 |
| <https://saskchiro.ca/> | Chiropractors’ Association of Saskatchewan | 2018 |
| <https://www.chiropratique.com/en/home.html> | Association des chiropraticiens du Québec | 2019 |
| <https://manitobachiropractors.ca/> | Manitoba Chiropractors Association | 2019 |
| <http://nlchiropractic.ca/> | Newfoundland and Labrador Chiropractic Association | 2018 |
| <https://www.peichiropractic.ca/> | Prince Edward Island Chiropractic Association | 2019 |
| <https://www.nbchiropractic.ca/> | New Brunswick Chiropractors Association | 2018 |
| <http://knowyourback.ca/> | Nova Scotia College of Chiropractors | 2019 |
| <https://www.cco.on.ca/> | College of Chiropractors of Ontario | 2018 |
| <http://www.chirobc.com/> | College of Chiropractors of British Columbia | 2018 |
| <http://www.chirofed.ca/english/index.html> | Federation of Canadian Chiropractic | 2012 |
| <https://allianceforchiropractic.com/> | Alliance for Chiropractic | 2018 |
| <http://www.rccssc.ca/> | Royal College of Chiropractic Sports Sciences | 2018 |
| <http://www.scca.ca/> | Student Canadian Chiropractic Association | 2016 |
| <https://www.canadianchiropracticresearchfoundation.ca/> | Canadian Chiropractic Research Foundation | 2018 |
| <http://www.cceb.ca/> | Canadian Chiropractic Examining Board | 2019 |
| <https://ccpaonline.microsoftcrmportals.com/en-US/> | Canadian Chiropractic Protective Association | 2018 |
| Naturopathic organizations | | |
| <https://www.cand.ca/> | Canadian Association of Naturopathic Doctors | 2018 |
| <https://oand.org/> | Ontario Association of Naturopathic Doctors | 2019 |
| <https://www.bcna.ca/> | British Columbia Naturopathic Association | 2019 |
| <http://www.collegeofnaturopaths.on.ca/> | The College of Naturopaths of Ontario | 2019 |
| <http://www.cnpbc.bc.ca/> | College of Naturopathic Physicians of British Columbia | 2019 |
| <https://www.cnda.net/> | College of Naturopathic Doctors of Alberta | 2018 |
| <https://www.anpq.qc.ca/en> | L’Association des Naturopathes Professionnells du Québec | 2019 |
| <https://ritma.ca/ritma-association.php> | The Ritma Association | 2019 |
| <https://www.anqnaturo.ca/en> | L’Association des Naturothérapeutes du Québec | 2019 |
| <https://www.naturopathie.ca/> | National Association of Naturopaths | 2019 |
| <http://qanm.org/> | Quebec Association of Naturopathic Medicine | 2017 |
| <https://www.sanp.ca/index.html> | Saskatchewan Association of Naturopathic Practitioners | 2014 |
| <https://nsand.ca/> | Nova Scotia Association of Naturopathic Doctors | 2019 |
| <https://www.nband.ca/> | New Brunswick Association of Naturopathic Doctors | n/a |
| <http://www.peiand.com/> | Prince Edward Island Association of Naturopathic Doctors | n/a |
| <https://www.cndmb.org/> | Manitoba Naturopathic Association | 2019 |
| <https://www.nhpcanada.org/index.html> | Natural Health Practitioners of Canada | 2017 |
| <http://apnn.ca/en> | Alliance of Professional Naturopaths and Naturotherapists | 2017 |
| Homeopathic organizations | | |
| <http://www.csoh.ca/> | Canadian Society of Homeopaths | 2019 |
| <http://www.collegeofhomeopaths.on.ca/> | College of Homeopaths of Ontario | 2018 |
| <http://www.wchs.info/> | West Coast Homeopathic Society | 2019 |
| <https://homeopathymanitoba.ca/> | Manitoba Homeopathic Association | 2018 |
| <https://bchomeopathy.ca/> | BC Association of Homeopaths | 2018 |
| <https://homeopathy.org/> | North American Society of Homeopaths | 2019 |
| <https://www.theohma.com/?fbclid=IwAR2gfOQo2vQzr3Ai09eDisFaOd75mOEO0OyT4U4bGt1U09gWjaatMa2P9Ic> | Ontario Homeopathic Medical Association | 2019 |
