# Supplementary material for: Vaccination discourses among chiropractors, naturopaths and homeopaths: A qualitative content analysis of academic literature and Canadian organizational webpages
Source: PLoS One. 2020 Aug 12;15(8):e0236691. doi: 10.1371/journal.pone.0236691 (PMC7423113; doi:10.1371/journal.pone.0236691)
Supplement: S3 Table — (DOCX) [file pone.0236691.s003.docx]

| Citation | Article sourced from reputable, peer-reviewed journal? | Publisher and country of origin | Institutional affiliation and/or location of first author at time of publication | Study/article purpose | Article type, methodology and/or methods |
| --- | --- | --- | --- | --- | --- |
| Adler, U. C. (2005). The influence of childhood infections and vaccination on the development  of atopy: A systematic review of the direct epidemiological evidence. Homeopathy, 94 (3), 182-195. | Yes | Thieme Medical Publishers, Germany | Alameda dos Jurupis, São Paulo, Brazil | Evaluate the veracity of the ‘hygiene hypothesis’ – that acquiring infectious disease prevents subsequent chronic allergic disease | Qualitative systematic review |
| Author unknown. (2007). The Flu: Sorting Fact from Fiction. Journal of Vertebral Subluxation Research (JVSR), 1-3. | Yes | McCoy Press, United States | Unknown | Discuss evidence pertaining to the safety and effectiveness of influenza vaccinations | Commentary/editorial/opinion |
| Bellavite, P. (2012). On the plausibility of homeopathic similitude. *Bioethics*, *26* (9), 506-7. | Yes | John Wiley & Sons, United States | Department of Scienze Morfologico-Biomediche, University of Verona, Verona, Italy | Address the theoretical plausibility and empirical evidence in support of the homeopathic principle of similitude | Commentary/editorial/opinion |
| Bellavite, P., Ortolani, R., Pontarollo, F., Pitari, G., Conforti, A. (2007). Immunology and  homeopathy. 5. The rationale of the ‘simile’. Evidence-based Complementary and Alternative Medicine, 4 (2), 149-63. | Yes | Hindawi Publishing Corporation, United Kingdom | Department of Pathology and Diagnostics, University of Verona, Verona, Italy | Describe the physiologic mechanisms and pharmacodynamics of homeopathic treatments | Narrative review; methods not specified |
| Birch, K. (2009). Notes on acute disease, genus epidemicus, and excerpts from vaccine free  prevention and treatment of infectious contagious disease with homeopathy. *Homoeopathic Heritage*, *34* (10), 28-32. | Undetermined | Bjain, India | Minnesota, United States | Examine the environmental, evolutionary and philosophic implications of epidemic-level disease outbreaks, as well as the role of homeopathic preparations in addressing epidemics | Commentary/editorial/opinion |
| Birch, K., & Whatcott, C. (2013). Homeoprophylaxis for the Prevention of Infectious Contagious  Disease. *Homoeopathic Links, 26* (2), 130-4 | Undetermined | Thieme Medical Publishers, Germany | Minnesota, United States | Review the current status of vaccination and homeoprophylaxis and posit why homeoprophylaxis is an appropriate alternative to vaccinartion | Narrative review; methods not specified |
| Bongiorno, P. B., & LoGiudice, P. (2004). Naturopathic medicine is indeed legitimate, effective, and wanted. *MedGenMed Medscape General Medicine, 6*(1). | Yes | Medscape, United States | New York City, New York, United States | Rebut claims that naturopathic medicine is ineffective | Letter to the editor |
| Busse, J. W., Morgan, L., & Campbell, J. B. (2005). Chiropractic antivaccination arguments. *Journal of Manipulative and Physiological Therapeutics, 28*(5), 367-73. | Yes | Mosby, United States | Department of Clinical Epidemiology and Biostatistics, McMaster University, Hamilton, Ontario, Canada | Review anti-vaccination arguments in chiropractic organization statements, publications and professional tradition | Narrative review; methods not specified |
| Cooperstein, R. (2008). Vaccination is both beyond and consistent with chiropractic philosophy! *Journal of the American Chiropractic Association, 45*(9), 27-8. | Yes | American Chiropractic Association, United States | Palmer College of Chiropractic West, San Jose, California, United States | Discuss how vaccination converges and diverges from foundational chiropractic philosophy | Commentary/editorial/opinion |
| Crump, S. C., & Oxley, M. (2003). Society of Homeopaths does not advise against vaccination. *British Medical Journal, 326*( 7381), 164-5. | Yes | BMJ, United Kingdom | Society of Homeopaths, Northampton, United Kingdom | Respond to evidence indicating a portion of homeopaths and chiropractors advise against vaccination. | Letter to the editor |
| Dehmeri, Z. S. (2012). Homeopathy: for adverse effects of vaccination. *Homoeopathic Heritage, 35* (11), 38-40. | Undetermined | Bjain, India | Gamadia Parsi Colony, Tardeo, Mumbai, India | Describe the uses of homeopathic preparations in remedying vaccine-related adverse events | Narrative review; methods not specified |
| Domalain, M. N., Peyrefitte, F., & Poitevin, B. (2010). Reflections on the homeopathic dilutions of vaccines. *Revue d'Homeopathie, 1* (3), 103-4. | Yes | Elsevier, The Netherlands | Malakof, Paris, France | Provide an overview of homeopathic prophylaxis and describe when it should be used | Commentary/editorial/opinion |
| Dunn, D., & Fior, T. (2014). A Modern-day Mad Hatter. *American Journal of Homeopathic Medicine, 107* (1), 25-34. | Yes | American Institute of Homeopathy, United States | National University of Health Sciences, Lombard, Illinois, United States | Examine case study of a patient suffering from various ailments stemming from chronic toxic mercury exposure | Narrative review with unspecified methods, case study |
| Eizayaga, J. E., & Waisse, S. (2016). What do homeopathic doctors think of vaccines? An international online survey. *Homeopathy, 105* (2), 180-5. | Yes | Thieme Medical Publishers, Germany | Department of Homeopathy, Maimonides University, Buenos Aires, Argentina | Investigate attitudes towards vaccination of Spanish- and Portuguese-speaking medically qualified homeopaths | Empirical, quantitative, online surveys, descriptive and inferential statistics |
| Eskinazi, D. (2005). Vaccinations: for or against. *Homeopathy, 94* (4), 252-3. | Yes | Thieme Medical Publishers, Germany | State University of New York, Stony Brook, New York, United States | Address conflicting research findings concerning adverse events following vaccination. | Commentary/editorial/opinion |
| Evans Jr, M. W., Perle, S. M., & Ndetan, H. (2011). Chiropractic wellness on the web: The content and quality of information related to wellness and primary prevention on the Internet. *Chiropractic and Manual Therapies, 19* (4), 1-7. | Yes | BioMed Central, United Kingdom | Texas Chiropractic College, Pasadena, Texas, United States | Evaluate the quality of the information presented on web pages produced through searches of “chiropractic wellness” based on standards for responsible health and medical information established by the Health on the Net foundation. | Empirical, quantitative, observational, content analysis, descriptive and analytic statistics |
| Ferrance, R. J. (2002). Vaccinations: how about some facts for a change? *Journal of the Canadian Chiropractic Association, 46* (3), 167-72. | Yes | Canadian Chiropractic Association, Canada | Tappahannock, Virginia, United States | Review major chiropractic organizations’ positions on vaccination, detail the evidence in favour and against vaccination, and advocate chiropractic embrace the practice | Commentary/editorial/opinion |
| Ferrance, R. J. (2003). Autism – another topic often lacking facts when discussed within the chiropractic profession. *Journal of the Canadian Chiropractic Association, 47* (1), 4-7. | Yes | Canadian Chiropractic Association, Canada | Tappahannock, Virginia, United States | Review evidence addressing the link between vaccines and autism | Commentary/editorial/opinion |
| Gleberzon, B.J., Lameris, M., Schmidt, C., & Ogrady, J. (2013). On vaccination & chiropractic: when ideology, history, perception, politics and jurisprudence collide. *Journal of the Canadian Chiropractic Association, 57* (3), 205-13. | Yes | Canadian Chiropractic Association, Canada | Canadian Memorial Chiropractic College,  Toronto, Ontario, Canada | Explore the issue of chiropractic and vaccination as it relates to historical perspectives, attitudes from chiropractic students and chiropractors, political perspectives, and issues of jurisprudence. | Narrative review; methods not specified |
| Gleberzon, B.J. & Rosenberg-Gleberzon, A.L. (2001). On autism: its prevalence, diagnosis, causes, and treatment. *Topics in Clinical Chiropractic, 8* (4), 42-69. | Yes | Aspen Publishers, United States | Canadian Memorial Chiropractic College  Toronto, Ontario, Canada | Review the diagnostic strategies, distribution, determinants and treatment options for autism spectrum disorders | Narrative review with vaguely stated methods |
| Golden, I. (2007). Vaccine damage -- prevention and treatment with homoeopathy. *American Journal of Homeopathic Medicine, 100* (2), 120-4. | Yes | American Institute of Homeopathy, United States | Australia | Summarize available evidence concerning vaccine safety and the effectiveness of homeopathic solutions in remedying vaccine-related adverse events | Narrative review; methods not specified |
| Golden, I. (2014a). The immunisation dilemma. *Homoeopathic Links, 27* (1), 11-15. | Undetermined | Thieme Medical Publishers, Germany | School of Science, Information Technology and Engineering, University of Ballarat, Victoria, Australia | Consider contemporary issues concerning medical immunization, discuss evidence in support of the effectiveness of homeopathic immunization | Narrative review; methods not specified |
| Golden, I. (2014b). The immunisation dilemma -- part two. *Homoeopathic Links, 27* (2), 74-8. | Undetermined | Thieme Medical Publishers, Germany | School of Science, Federation University, Victoria, Australia | Review the long-term safety of medical immunization and homeoprophylaxis | Narrative review; methods not specified |
| Golden, I. (2014). The philosophical and evidentiary basis of homoeopathic immunisation: a response to Teixeira. *International Journal of High Dilution Research, 13* (46), 45-53. | Yes | Universidade Estadual Paulista – UNESP, Brazil | School of Science, Federation University, Victoria, Australia | Rebut another author’s claim that nosodes are an ineffective form of homeopathic prophylaxis | Narrative review; methods not specified |
| Golden, I. (2015a). The immunisation dilemma--part 5: towards international agreement on homoeoprophylaxis. *Homoeopathic Links, 28*(2), 75-80. | Undetermined | Thieme Medical Publishers, Germany | School of Science, Federation University, Victoria, Australia | Offer a majority position on homeoprophylaxis among homeopaths based on previous research findings | Commentary/editorial/opinion |
| Golden, I. (2015b). The immunisation dilemma--part four: a comparison of effectiveness. *Homoeopathic Links, 28*(1), 8-13. | Undetermined | Thieme Medical Publishers, Germany | School of Science, Federation University, Victoria, Australia | Examine the type and quality of evidence supporting the safety and effectiveness of both homeoprophylaxis and vaccination | Described as systematic review, methods not explicitly stated |
| Golden, I. (2016a). An example of issues of editorial bias confronting questioning researchers. *Homoeopathic Links, 29* (2), 117-9. | Undetermined | Thieme Medical Publishers, Germany | School of Science, Federation University, Victoria, Australia | Detail a series of interactions between research teams and editorial staff following publication of an article in *Vaccine.* | Commentary/editorial/opinion |
| Golden, I. (2016b). The immunisation dilemma--part 6: towards agreement on homeoprophylaxis. *Homoeopathic Links, 29*(2), 147-9. | Undetermined | Thieme Medical Publishers, Germany | School of Science, Federation University, Victoria, Australia | Summarize findings from previous articles pertaining to attitudes regarding homeoprophylaxis among homeopaths | Narrative review; methods not specified |
| Golden, I., & Stranieri, A. (2014). The immunisation dilemma -- part 3.1. *Homoeopathic Links, 27* (3), 171-9. | Undetermined | Thieme Medical Publishers, Germany | School of Science, Federation University, Victoria, Australia | Assess attitudes towards and use of homoeoprophylaxis among accredited homeopaths. | Empirical, quantitative, observational, surveys, descriptive and inferential statistics |
| Golden, I., Stranieri, A., & Nuaimat, A.A, (2014). The immunisation dilemma – part 3.2. *Homoeopathic Links, 27* (4): 190-6. | Undetermined | Thieme Medical Publishers, Germany | School of Science, Federation University, Victoria, Australia | Assess attitudes towards and use of homoeoprophylaxis among accredited homeopaths | Empirical, quantitative, observational, surveys, descriptive and inferential statistics |
| Halperin, S. A., Pless, R. (2003). Immunization in Canada: a success to build on. *Journal of the Canadian Chiropractic Association, 47* (3), 153-160. | Yes | Canadian Chiropractic Association, Canada | Departments of Pediatrics, and Microbiology and Immunology, Dalhousie University and the IWK Health Centre, Halifax, Nova Scotia, Canada | Review current status of vaccines and vaccine-preventable illness, contextualize the risks and benefits of vaccination | Commentary/editorial/opinion |
| Hiltner, R. (2014). Fifteen years' experience with homeopathic immunizations (homeoprophylaxis). *American Journal of Homeopathic Medicine, 107* (2), 60-3. | Yes | American Institute of Homeopathy, United States | Ojai, California, United States | Evaluate the effectiveness of homeoprophylaxis in preventing the emergence of Polio, Diphtheria, Pertussis and Tetanus | Non-systematic experimental protocol. No random assignment, no baseline assessment, no control group |
| Homola, S. (2001). Is the chiropractic subluxation theory a threat to public health? Symposium on 'alternative' public health threats. *Scientific Review of Alternative Medicine, 5* (1): 45-53. | Yes | Prometheus Books Inc., United States | Panama City, Florida, United States | Review uptake of vertebral subluxation theory in chiropractic and explore its impact on public health | Narrative review; methods not specified |
| Jacobs, J. (2018). Homeopathic prevention and management of epidemic diseases. *Homeopathy, 107* (3): 157-60. | Yes | Thieme Medical Publishers, Germany | Department of Epidemiology, School of Public Health and Community Medicine, University of Washington, Seattle, Washington, United States | Review evidence of the effectiveness of various homeopathic strategies for the treatment and prevention of illness | Narrative review; methods not specified |
| Johnson, C., Baird, R., Dougherty, P. E., Globe, G., Green, B. N., Haneline, M., . . . Smith, M. (2008). Chiropractic and public health: current state and future vision. *Journal of Manipulative & Physiological Therapeutics, 31* (6), 397-410. | Yes | Mosby, United States | Southern California University of Health Sciences. Whittier, California, United States | Document chiropractic’s involvement in the public health movement, reflect on past barriers that may have prevented participation in public health initiatives, and examine the relationship between chiropractic and current public health topics. | Editorial, collaborative summary, narrative review |
| Johnson, C., Rubinstein, S. M., Cote, P., Hestbaek, L., Injeyan, H. S., Puhl, A., . . . Kopansky-Giles, D. R. (2012). Chiropractic care and public health: Answering difficult questions about safety, care through the lifespan, and community action. *Journal of Manipulative and Physiological Therapeutics, 35* (7), 493-513. | Yes | Mosby, United States | National University of Health Sciences, Lombard, Illinois, United States | Document chiropractic’s involvement in public health and summarize its relationship with public health topics of safety, health through the lifespan, and effective participation in community health issues. | Editorial, collaborative summary, narrative review |
| Kent, C. & Gentempo, P. (1990). Immunization: Facts, myths and speculation. *ICA International Review of Chiropractic*, 13-21. | Yes | International Chiropractors Association, United States | San Jose, California, United States | Disambiguate fact from myth regarding vaccine safety and effectiveness | Commentary/editorial/opinion |
| Khorsan, R., Smith, M., Hawk, C., & Haas, M. (2009). A public health immunization resource web site for chiropractors: Discussion of current issues and future challenges for evidence-based initiatives for the chiropractic profession. *Journal of Manipulative and Physiological Therapeutics, 3 2*(6), 500-4. | Yes | Mosby, United States | University of California Irvine, Irvine, California, United States | Describe the Immunization Information Resource Website, developed and sponsored by the Chiropractic Health Care Section of the American Public Health Association, as well as discussing current and future challenges advancing similar evidence-based initiatives. | Commentary/editorial/opinion |
| Lanfranchi, R.G. (1993). Vaccinations: think before you embrace. *ICA International Review of Chiropractic*, 43-9. | Yes | International Chiropractors Association, United States | New York, United States | Review data concerning vaccine safety and effectiveness | Narrative review; methods not specified |
| Lee, N. (2000). Breast feeding should be promoted. *BMJ, 321* (7253), 108. | Yes | BMJ, United Kingdom | Sandwich, Massachusetts, United States | Respond to claims in Bedford and Elliman’s (2000) article that emphasized the safety and effectiveness of vaccination | Letter to the editor |
| Mackay, J. (2012). The history of homeo-prophylaxis and review of the evidence to support its efficacy. *Homoeopathic Links, 25* (1), 56-61. | Undetermined | Thieme Medical Publishers, Germany | Taplow, Berkshire, United Kingdom | Review historical positions on homeoprophylaxis and recent evidence concerning its safety and effectiveness | Narrative review; methods not specified |
| Master, F. J. (2012). Vaccination and homeopathy. *Homoeopathic Heritage, 35* (11), 9-11. | Undetermined | Bjain, India | Mumbai, India | Offer a homeopathic perspective on vaccination | Commentary/editorial/opinion |
| McCoy, M. (2008). Autonomy, consent, and medical paternalism: Legal issues in medical intervention. *Journal of Alternative and Complementary Medicine, 14* (6), 786-92. | Yes | Mary Ann Liebert, United States | Life University, Marietta, Georgia, United States | Review issues of autonomy, informed consent and medical paternalism as they pertain to mandatory vaccination and forced cancer treatment | Narrative review; methods not specified |
| Morgan, L.G. (1997). Pertussis immunization: an update. *Journal of the Canadian Chiropractic Association, 41* (2), 86-90. | Yes | Canadian Chiropractic Association, Canada | Nampa, Idaho, United States | Review evidence pertaining to the safety and effectiveness of pertussis immunization | Narrative review; methods not specified |
| Morrel, P. (2000). Is vaccination cause célèbre or bête noire? *BMJ, 321* (7253), 108-9. | Yes | BMJ, United Kingdom | Department of Sociology, Staffordshire University, Stoke-on-Trent, England, United Kingdom | Respond to claims in Bedford and Elliman’s (2000) article that emphasized the safety and effectiveness of vaccination | Letter to the editor |
| Moskowitz, R. (2009). An autistic boy. *American Journal of Homeopathic Medicine, 102* (3), 117-21. | Yes | American Institute of Homeopathy, United States | Watertown, Massachusetts, United States | Detail a case study of an individual with autism whose symptoms purported worsened following MMR vaccination | Case study |
| Moskowitz, R. (2010). Vaccines, drugs, and other causes: a homeopath looks at the medical system: part one. *American Journal of Homeopathic Medicine, 103*(4), 214-7. | Yes | American Institute of Homeopathy, United States | Watertown, Massachusetts, United States | Detail the philosophic position of evidence-based medicine, or lack thereof. | Commentary/editorial/opinion |
| Moskowitz, R. (2011). Vaccines, Drugs, and Other Causes: A Homeopath Looks at the Medical System. Part 2. American Journal of Homeopathic Medicine, 104(1), 13-26. | Yes | American Institute of Homeopathy, United States | Watertown, Massachusetts, United States | Posit reasons as to why vaccine-related adverse events are under-emphasized among practitioners and institutions of evidence-based medicine | Commentary/editorial/opinion |
| Moskowitz, R. (2017). Vaccine-related illness. *American Journal of Homeopathic Medicine, 110* (2), 35-40. | Yes | American Institute of Homeopathy, United States | Watertown, Massachusetts, United States | Posit reasons as to why vaccine-related adverse events are under-emphasized among practitioners and institutions of evidence-based medicine | Commentary/editorial/opinion |
| Nelson, C. (1993). Why chiropractors should embrace immunization. *ACA Journal of Chiropractic, 30*: 88-95. | Yes | American Chiropractic Association, United States | Northwestern College of Chiropractic, Bloomington, Minnesota, United States | Describe and respond to common anti-vaccination arguments among chiropractors | Commentary/editorial/opinion |
| Rafeeque, M. (2012). An attempt to solve the controversies in homeopathic prophylaxis. *Homoeopathic Heritage, 35* (11), 32-7. | Undetermined | Bjain, India | Department of Homeopathy, Government of Kerala, Kerala, India | Consult existing evidence to resolve controversies on homeoprophylaxis | Narrative review with unspecified methods supplemented with survey data |
| Roy, R. (2016). Protection against diseases using homeopathy. *Homoeopathic Links, 29* (2), 101-3. | Undetermined | Thieme Medical Publishers, Germany | Riegsee, Germany | Outline the philosophic and professional mandate of homeopathy concerning illness prevention | Commentary/editorial/opinion |
| Rozencwajg, J. (2015). There is a choice homeoprophylaxis--an appeal to mothers. *Homoeopathic Links, 28* (2), 135. | Undetermined | Thieme Medical Publishers, Germany | New Zealand | Review Whatcott’s (2015) text | Book review |
| Saltzman, S. (2016). Shining light on the darkness. *American Journal of Homeopathic Medicine, 109*, 37. | Yes | American Institute of Homeopathy, United States | Hartsdale, New York, United States | Discuss the importance of being cognizant to the role the medical-industrial complex plays in generating vaccine-induced autism | Commentary/editorial/opinion |
| Saxton, J. G. (2005). Do we truly understand vaccine reactions and vaccinosis? *Homeopathy, 94* (3), 200-1. | Yes | Thieme Medical Publishers, Germany | Leeds, United Kingdom | Define vaccinosis, distinguish vaccinosis from acute vaccine reactions, detail the clinical progression of vaccinosis | Commentary/editorial/opinion |
| Scheibner, V. (2003). Response to Leask and McIntyre’s attack on myself as a public opponent of vaccination. Vaccine, 22 (1), vi-ix. | Yes | Elsevier, United States | New South Wales, Australia | Respond to comment from other authors | Letter to the editor |
| Singh, A. (2008). Vaccine reaction. *Homoeopathic Heritage, 33* (11), 15-8. | Undetermined | Bjain, India | Kolkata, India | Comment on the safety of vaccines and their consistency with homeopathic principles | Commentary/editorial/opinion |
| Smits, T. (2007). Post-vaccinal behavioural problems: ill humour, anxiety, aggressiveness, contact disturbances, hysterical behaviour, ADHD, PDD-nos, ADD, autism, cured with potentised vaccines. *Homoeopathic Links, 20* (2), 93-9. | Undetermined | Thieme Medical Publishers, Germany | Waalre, The Netherlands | Demonstrate the links between behavioural problems, autism, and vaccination, as well as prove that homeopathic remedies can cure vaccine-related illness | Case series of 30 children displaying behavioural problems following vaccination |
| Teixeira, M.Z. (2002). Is there scientific evidence that suppression of acute diseases in childhood induce chronic diseases in the future? *Homeopathy, 91* (4), 207-16. | Yes | Thieme Medical Publishers, Germany | Department of Clinical Medicine, Faculty of Medicine, Universidade de São Paulo, São Paulo, Brazil | Review evidence of vaccine-related acute and chronic illness, propose a mechanism for vaccine-related pathophysiology | Narrative review; methods not specified |
| Teixeira, M.Z. (2009). Homeopathy: a preventive approach to medicine? . *International Journal of High Dilution Research, 8* (29), 155-72 | Yes | Universidade Estadual Paulista – UNESP, Brazil | Department of Clinical Medicine, Faculty of Medicine, Universidade de São Paulo, São Paulo, Brazil | Review classic homeopathic writings and empirical evidence to evaluate homeopathy’s effectiveness for treatment and prevention | Narrative review with vaguely stated methods |
| Thompson, T. (2002). CAM before the storm -- alternative medicine and the great MMR debate. *Complementary Therapies in Medicine, 10* (2), 126-7. | Yes | Elsevier, United States | Division of Primary Health Care, Department of Clinical Medicine, Canynge Hall, Whiteladies Road, Bristol, England, United Kingdom | Review vaccine discourses from the webpages of CAM organizations and prominent CAM providers | Narrative review with vaguely stated methods |
| Vernon, L. F., & Kent, C. (2009). Chiropractors and vaccinations: Ethics is the real issue. *Complementary Health Practice Review, 14* (1), 36-50. | Yes | Springer Publishing, United States | Bridgeport, Connecticut, United States | Detail the historical and philosophical evolution of pro- and anti-vaccination arguments, particularly with regard to chiropractic. | Narrative review; methods not specified |
| Vithoulkas, G. (2008). British media attacks on homeopathy: Are they justified? *Homeopathy, 97* (2), 103-6. | Yes | Thieme Medical Publishers, Germany | International Academy of Classical Homeopathy, Alonissos, Greece | Explain why eccentric views from fringe practitioners are fueling anti-homeopathic attacks from mainstream media | Commentary/editorial/opinion |
| Wiese, G. (1996). Chiropractic's tension with the germ theory of disease. *Chiropractic History, 16* (1), 72-87. | Yes | Association for the History of Chiropractic, United States | Palmer College of Chiropractic, Davenport, Iowa, United States | Review the evolution of chiropractic’s position regarding the germ theory of disease and medical interventions based thereupon. | Narrative review; methods not specified |
| Yasgur, J. (2018). Vaccines: a reappraisal. *Homoeopathic Links, 31* (1), 86. | Undetermined | Thieme Medical Publishers, Germany | United States | Review Moskowitz’s (2017) text | Book review |
