# Supplementary material for: Vaccination discourses among chiropractors, naturopaths and homeopaths: A qualitative content analysis of academic literature and Canadian organizational webpages
Source: PLoS One. 2020 Aug 12;15(8):e0236691. doi: 10.1371/journal.pone.0236691 (PMC7423113; doi:10.1371/journal.pone.0236691)
Supplement: Attachment — Submitted filename: Response to reviewers for PLoS - vaccine CAM.docx [file pone.0236691.s006.docx]

| Editor/reviewer comment | Authors’ response |
| --- | --- |
| Editor | |
| Reviewer 1 has made some comments which are debatable. You do not exclusively cite opinions unfavorable to vaccination, and you do separate your discussion of academic literature from other sources. Nevertheless you should consider why the reviewer responded in this way and make the rationale for what you have done more clear. | We appreciate the editor’s acknowledging our effort to faithfully represent the diversity of vaccine attitudes, both pro- and anti-, in the CAM literature. With due respect to reviewer 1, we made a point of being as thorough and transparent as possible in describing our methods of data analysis and quote selection. On page 8, we explicitly note, “In choosing quotes to illustrate our themes, we made our best effort to provide a balanced and diverse array of viewpoints across various disciplines, authors, publication types and stances on any given issue while also ensuring the quotes selected most accurately exemplify the themes being articulated.” We have provided greater clarity about the aim and scope of our paper in our revised manuscript (discussed below) in the case that other readers share the concerns of reviewer 1. |
| The criticism of one journal that you cite in the discussion is legitimate but you should more fully describe the source, as would be helpful to the reader in many other instances. | Reviewer 1’s response suggests to us that some readers might view our statement of the journal Homeopathy’s scientific status as too polemical. Normally we would address such concerns by further fleshing out our argument with additional supporting evidence. In this case, however, given the argument is tangential to our central empirical claims (i.e. we note inquiry into the quality of CAM research is extraneous to our objectives), as well as the editor’s request to shorten the manuscript, we thought it more prudent to omit the statement outright. We still present these specific journals as “undetermined” in relation to whether they are peer-reviewed, and as we discuss below, provide greater explanation for our approach in identifying them as so. We feel this information is important for readers in their interpretation of source content and arguments put forth by undetermined sources. |
| I believe there is substantial controversy about the standards of some CAM journals and their standards of evidence, which you might want to explore more deeply. While I understand that your purpose is to present what you find out in the world, and not criticism, I don't think you need to be entirely agnostic about the quality of evidence in the academic, or in some cases questionably academic literature that you review. You state that a full inquiry into the quality of this evidence is a fit subject for future inquiry but you could better emphasize the importance of the question. | We have expanded on our method for establishing the peer-reviewed status of articles and our decision to include articles contained in “undetermined” journals (p. 34). In an effort to be less agnostic while still maintaining an appearance of objectivity, we encourage readers to consider the veracity of authors’ claims in light of the ambiguity in their peer-reviewed status (p. 34). |
| Also, please pay attention to Reviewer 2's comments. What appears on the web sites of the associations is not necessarily informative about how CAM practitioners actually advise clients. What you find in the published literature suggests to me that there is more skepticism among practitioners than the official guidance would indicate. | We agree this is an important point. The grant funding this paper is more broadly investigating the nature and extent of vaccine beliefs and CAM practice. However, the present paper, the first phase of this larger project, seeks only to characterize vaccine-related discourses in the CAM literature. It is very important to us that these issues are not conflated and we’ve therefore made it very clear in the manuscript what our focus was in the design of this project.  On page 5 we state, “At the outset, we wish to clarify the findings presented herein do not necessarily reflect the knowledge, attitudes and practices of CAM providers in general; we seek only to characterize the vaccine-related views promulgated by sources that putatively inform CAM practice.”  Similarly, on page 32 we note, “First, we wish to reiterate that any points made with regard to vaccine attitudes in the CAM literature and organizational webpages are not intended to be generalizable to chiropractors, naturopaths and homeopaths by and large. Although this review qualitatively describes professional discourses that may influence practitioners’ vaccine-related knowledge, attitudes and practices, it is outside the scope of this study to determine precisely how and to what extent these socio-professional influences are actually reflected among practitioners “in the field”.”  We are aware of the evidence of an association between CAM use and vaccine refusal or hesitancy (and indeed cite this literature). However, we do not have evidence to suggest that CAM providers are fuelling that hesitancy in their consultations. An investigation into how these vaccine beliefs and attitudes are reflected in CAM providers’ actual practice is a separate research question that we aim to answer in through interviews with CAM providers. |
| Reviewer 1 also refers to controversy among CAM advocates and practitioners, which you might want to describe more fully. | See above comment on providers’ vaccine-related practices being extraneous to our study objectives, but an important avenue for future inquiry. To further develop this point, we have added evidence of CAM providers’ diverging vaccine-related attitudes and practices to underscore that the views and prescriptions we detail likely do not elicit total support or compliance (p. 33). |
| I agree that the term "allopathic" medicine is questionable. This term arose historically to describe a school of medicine in opposition to homeopathy. However, that is no longer definitional of what is taught in medical school and ascribes too much status to homeopathy as an equally plausible alternative. It would be better to say science based medicine, or perhaps standard medical practice. | All instances of the term “allopathic medicine” have been replaced with “evidence-based medicine.” |
| Finally, I agree with both reviewers that you should try to shorten this considerably. | We appreciate the editor’s concern regarding manuscript length. To address this, Tables 2 and 4 have been moved to supplementary files, cutting some 3,500 words. We have already made our best attempt at paring back the main body of text, and fear that to do so further would compromise the persuasiveness of our arguments or our ability to properly represent the rigour of our methodology. |
| Please ensure that your manuscript meets PLOS ONE's style requirements, including those for file naming. | Figure 1 file has been renamed to Fig1.tiff. |
| Could you therefore please include the title page into the beginning of your manuscript file itself, listing all authors and affiliations. | Title page has been added to the manuscript file. |
| Reviewer 1 | |
| There is too much heterogeneity among sources to grant joint analysis: papers in peer-reviewed, PubMed and JCR indexed journals are analysed together with blogs, opinions, and even comments by non-practitioners! | As the editor acknowledges, our use of external sources is compartmentalized such that analysis is restricted to prima facie peer-reviewed articles and organizational webpages, while citations of other sources are reserved for the discussion. |
| It's not clear why several papers were included for analysis, nor why you "randomly selected 30 from 65". | Selecting and analyzing a random sample from a dataset is common practice in quantitative research, particularly when the dataset is prohibitively large -- in this case, 1921 citations across 65 articles – and/or when analyses cannot be performed computationally. |
| There is bias in the presentation of the results: only opinions against vaccines were described, and you fully omitted discussions within communities of practitioners. | Please see the above response to editor. |
| In addition to "n" you should provide the reference for the citations, so readers can know how many of the included papers are actually cited. | We appreciate this request but citing every individual datum point that contributes to a particular theme is inconsistent with thematic analysis protocol. On a purely logistical note, it would also make reading the paper more unwieldy. We opted to retain frequency counts, despite contrariwise convention in qualitative research, to give readers a sense of themes’ “internal representativeness” – offering citations as well, we feel, does not further this aim. |
| The attempt to disqualify journal Homeopathy (peer-reviewed, PubMed and JCR indexed) using a blog (!) is uncalled for and makes explicit the bias that impregnates the entire Results and large part of the Discussion section/ | See above response to editor. |
| Reviewer 2 | |
| The paper is rather long, and it would be much better if the authors try to make it shorter. | See above response to editor. |
| We all know that despite the fact that CAM physicians do not have any guidelines against vaccines their attitude and practice is different. One of the results in our recent study (Vaccine confidence among parents: Large scale study in eighteen European countries) published in the vaccine journal is the following: Parents who consult with paediatricians or nurses had lower hesitancy scores than parents who consult either with GPs (15.4 vs. 23.1, p < 0.05) or with homeopathists (15.4 vs. 51.3, p < 0.05). So, we need to be very careful not to give the wrong message to the reader. | See above response to editor. |
